# Supplementary material for: College Students’ Degree of Support for Online Learning during the COVID-19 Pandemic and Associated Factors: A Cross-Sectional Study
Source: Int J Environ Res Public Health. 2022 Dec 14;19(24):16814. doi: 10.3390/ijerph192416814 (PMC9779361; doi:10.3390/ijerph192416814)
Supplement: Supplementary file 1 [file ijerph-19-16814-s001.zip › ijerph-2030131-supplementary.pdf]

Supplementary Materials File S1: Scales

Perceived Social Support Scale.

Please choose one option after each sentence based on how you perceive the actual social support	Extremely disagree	Strongly disagree	Slightly Disagree	General	Slightly Agree	Strongly Agree	Extremely Agree
1. There is a special person who is around when I am in need	1	2	3	4	5	6	7
2. There is a special person with whom I can share my joys and sorrows	1	2	3	4	5	6	7
3. My family really tries to help me	1	2	3	4	5	6	7
4. I get the emotional help and support I need from my family	1	2	3	4	5	6	7
5. I have a special person who is a real source of comfort to me	1	2	3	4	5	6	7
6. My friends really try to help me	1	2	3	4	5	6	7
7. I can count on my friends when things go wrong	1	2	3	4	5	6	7
8. I can talk about my problems with my family	1	2	3	4	5	6	7

9. I have friends with whom I can share my joys and sorrows	1	2	3	4	5	6	7
10. There is a special person in my life who cares about my feelings	1	2	3	4	5	6	7
11. My family is willing to help me make decisions	1	2	3	4	5	6	7
12. I can talk about my problems with my friends	1	2	3	4	5	6	7

领悟社会支持量表

请您根据自己感受到社会支持的实际情况，在每句后面选择一个答案	极不同意	很不同意	稍不同意	中立	稍同意	很同意	极同意
1. 在我遇到问题时，有些人 (亲戚、邻居、同事）会出现在我身旁	1	2	3	4	5	6	7
2. 我能与有些人（亲戚、邻居、同事）共享我的快乐与忧伤	1	2	3	4	5	6	7
3. 我的家庭能够切实解决我的问题	1	2	3	4	5	6	7
4. 在我需要时我能从家庭获得情感上的帮助和支持	1	2	3	4	5	6	7
5. 当我有困难时，有些人（亲戚、邻居、同事）是安慰我的真正源泉	1	2	3	4	5	6	7
6. 我的朋友们真正帮助我	1	2	3	4	5	6	7

7. 在发生困难时，我可以依靠我的朋友们	1	2	3	4	5	6	7
8. 我能与自己的家庭谈论我的难题	1	2	3	4	5	6	7
9. 我的朋友们能与我分享快乐与忧伤	1	2	3	4	5	6	7
10. 在我的生活中有些人（亲戚、邻居、同事）关心着我的感情	1	2	3	4	5	6	7
11. 我的家庭能心甘情愿的协助我做出各种决定	1	2	3	4	5	6	7
12. 我能与朋友们讨论自己的难题	1	2	3	4	5	6	7

Family Communication Scale

Please select the option that best matches your reality	Strongly disagree	Comparatively disagree	General	Comparatively agree	Strongly agree
1. Family members are satisfied with how they communicate with each other	1	2	3	4	5
2. Family members are very good listeners	1	2	3	4	5
3. Family members express affection to each other	1	2	3	4	5
4. Family members are able to ask each other for what they want	1	2	3	4	5
5. Family members can calmly discuss problems with each other	1	2	3	4	5
6. Family members discuss their ideas and beliefs with each other	1	2	3	4	5

7. When family members ask questions of each other, they get honest answers	1	2	3	4	5
8. Family members try to understand each other's feelings	1	2	3	4	5
9. When angry, family members seldom say negative things about each other	1	2	3	4	5
10. Family members express their true feelings to each other	1	2	3	4	5

家庭沟通量表

请选择最符合您现实状况的选项	强烈不同意	比较不同意	中立	比较同意	强烈同意
1. 家庭成员都对彼此间的沟通方式感到满意	1	2	3	4	5
2. 家庭成员们都是非常好的倾听者	1	2	3	4	5
3. 家庭成员彼此之间会互相表达爱意	1	2	3	4	5
4. 家庭成员之间可以互相询问彼此想要什么	1	2	3	4	5
5. 家庭成员们之间可以冷静地讨论问题	1	2	3	4	5
6. 家庭成员之间可以讨论彼此的想法和信仰	1	2	3	4	5
7. 当家庭成员之间提问时，他们会得到如实的回答	1	2	3	4	5
8. 家庭成员尝试去理解彼此的感受	1	2	3	4	5

9. 当生气时，家庭成员之间也很少说负面的话	1	2	3	4	5
10. 家庭成员彼此之间会表达真实感受	1	2	3	4	5

PHQ-9

Over the *last 2 weeks*, how often have you been bothered by any of the following problems?	Not at all	Several days	More than half the days	Nearly every day
1. Little interest or pleasure in doing things	0	1	2	3
2. Feeling down, depressed, or hopeless	0	1	2	3
3. Trouble falling or staying asleep, or sleeping too much	0	1	2	3
4. Feeling tired or having little energy	0	1	2	3
5. Poor appetite or overeating	0	1	2	3
6. Feeling bad about yourself—or that you are a failure or have let yourself or your family down	0	1	2	3
7. Trouble concentrating on things, such as reading the newspaper or watching television	0	1	2	3
8. Moving or speaking so slowly that other people could have noticed? Or the opposite—being so fidgety or restless that you have been moving around a lot more than usual	0	1	2	3
9. Thoughts that you would be better off dead or of hurting yourself in some way	0	1	2	3

9 项患者健康问卷

在过去两周里，你有多长时间被以下问题所困扰？	从来	有几天	刚超过一半天数	接近每天

	没有			
1. 做任何事都觉得沉闷或者根本不想做任何事	0	1	2	3
2. 情绪低落、抑郁或绝望	0	1	2	3
3. 难于入睡；半夜会醒，或相反，睡觉时间过多	0	1	2	3
4. 觉得疲倦或没有活力	0	1	2	3
5. 胃口极差或饮食过量	0	1	2	3
6. 不喜欢自己——觉得自己做得不好、对自己失望或有负家人期望	0	1	2	3
7. 难于集中精神做事，例如看报纸或看电视	0	1	2	3
8. 其它人反映你行动或说话迟缓；或者相反，您比平常活动更多——坐立不安、停不下来	0	1	2	3
9. 想到自己死或者自残	0	1	2	3

Three-Item Loneliness Scale

How often do you feel	Hardly Ever	Sometimes	Often
1. left out	1	2	3
2. isolated from others	1	2	3
2. lack companionship	1	2	3

孤独感三项量表

请选择下列情况出现的频率	几乎从不	有时	经常
1. 您感到被遗忘	1	2	3
2. 您感到与他人隔绝	1	2	3
2. 您觉得自己缺乏陪伴	1	2	3
